# Nephroprotection by SGLT2i in CKD Patients: May It Be Modulated by Low-Protein Plant-Based Diets?

**DOI:** 10.3389/fmed.2020.622593

**Published:** 2020-12-03

**Authors:** Adamasco Cupisti, Domenico Giannese, Diego Moriconi, Claudia D'Alessandro, Massimo Torreggiani, Giorgina B. Piccoli

**Affiliations:** ^1^Department of Clinical and Experimental Medicine, University of Pisa, Pisa, Italy; ^2^Néphrologie, Centre Hospitalier Le Mans, Le Mans, France; ^3^Dipartimento di Scienze Cliniche e Biologiche, Università di Torino, Torino, Italy

**Keywords:** CKD-chronic kidney disease, SGLT2 inhibitors, diabetic nephropathy, diet, low protein diet, plant based diet

## Abstract

Sodium-glucose-transporter 2 inhibitors (SGLT2i) are a new class of anti-diabetic drugs that in large trials such as CREDENCE have shown also a reduction of glomerular hyperfiltration and albuminuria in type 2 diabetic patients. Hence, the interest toward SGLT2i is focused toward this potential nephroprotective effect, in order to reduce the progression to overt nephropathy, and it seems to be confirmed in the most recent DAPA-CKD trial. This is the reason why the indication for SGLT2i treatment has been extended to chronic kidney disease (CKD) patients with eGFR up to 30 ml/min, namely with CKD stage 1–3. In patients with CKD stage 3 to 5, the most recent KDIGO guidelines recommend low-protein diet and plant-based regimens to delay end-stage kidney disease (ESKD) and improve quality of life. Similarly to SGLT2i, low-protein diets exert renal-protective effects by reducing single nephron hyperfiltration and urinary protein excretion. Beyond the glomerular hemodynamic effects, both protein restriction and SGLT2i are able to restore autophagy and, through these mechanisms, they may exert protective effects on diabetic kidney disease. In this perspective, it is likely that diet may modulate the effect of SGLT2i in CKD patients. Unfortunately, no data are available on the outcomes of the association of SGLT2i and low-protein and/or vegan diets. It is therefore reasonable to investigate whether CKD patients receiving SGLT2i may have further advantages in terms of nephroprotection from the implementation of a low-protein and/or plant-based diet or whether this association does not result in an additive effect, especially in vascular nephropathies.

## Introduction

Sodium-glucose-transporter 2 inhibitors (SGTL2i) are a new class of anti-diabetic drugs able to improve glycemic control. While this primary effect is undoubtedly interesting, the major point of interest in these drugs is related to their cardiovascular and renal protective properties. SGLT2i have been shown to reduce several cardiovascular endpoints in high-comorbidity patients with type 2 diabetes (1, 2). Of note, the CREDENCE trial was pre-maturely terminated at the ad interim analysis because of the evident benefits toward the primary composite endpoint of doubling of serum creatinine and renal or cardiovascular death, in treated patients (3).

Randomized controlled trials such as EMPAREG, CANVAS and DECLARE-TIMI, have shown that different SGLT2i (empagliflozin in EMPAREG, canagliflozin in CANVAS and dapagliflozin in DECLARE-TIMI) reduced glomerular hyperfiltration and albuminuria in type 2 diabetic patients (1, 3, 4). As a consequence, the interest toward SGLT2i shifted from their role in diabetes control to their potential to reduce the incidence of overt nephropathy (5). The randomized controlled trial CREDENCE was specifically designed to assess renal survival in a large cohort of type 2 diabetic patients with chronic kidney disease (CKD) and confirmed the favorable effect of canagliflozin on the risk of kidney failure (2). In fact, proteinuric patients with CKD stage 2 and 3 treated with canagliflozin showed a 30% decreased risk of reaching the composite end-point of end-stage kidney disease, doubling of serum creatinine levels or death for renal or cardiac causes over a media follow-up of 2.62 years. Moreover, the benefits of canagliflozin seemed to be greater in patients with worst kidney function and more severe proteinuria (2). It has also been reported that Dapagliflozin was able to improve endothelial function and arterial stiffness as well as renal resistive index, likely mediated by oxidative stress reduction in type 2 diabetics (6, 7).

The nephroprotective effect of SGLT2i has been mainly attributed to the modulation of glomerular hemodynamics, as we will discuss in detail in the next paragraphs.

The intrarenal effects of SGLT2 inhibitors aroused great interest which was initially limited to diabetic patients with increased, normal or only slightly reduced kidney function. More recently, accumulating evidences suggested testing these molecules in more advanced stages of CKD in the course of type 2 diabetes mellitus. The extraordinary results recorded in diabetic patients, independently of glycemic control, suggest today a role for SGLT2i even in non-diabetic nephropathies.

Recent data of the DAPA-CKD trial that included CKD patients with and without diabetes, suggested a significant protective effect of dapagliflozin on CKD outcomes (8). This beneficial effect seemed to be even greater in non-diabetic patients. Again, the DAPA-CKD study was terminated earlier based on the suggestion on an independent committee because of the superior efficacy compared to the placebo. A sub-analysis of the DAPA-HF study showed that, in patients with reduced ejection fraction hearth failure, dapagliflozin was able to reduce the progression of kidney disease in both diabetic and non-diabetic patients over a 2 year follow-up (9). Finally, the DIAMOND study showed no benefits on proteinuria in 53 non-diabetic CKD patients with a mean GFR of 58 ml/min, over a 6 week therapy with a cross-over design (10).

It appears that dapagliflozin, like other SGLT2i, can be useful also for non-diabetic patients, at least up to CKD stage 3. These data progressively extended canagliflozin prescription indications and it can now be prescribed to patients with a moderate reduction of renal function with an estimated glomerular filtration rate (eGFR) up to 30 ml/min/1.73 m^2^; furthermore, once started at an eGFR > 30 ml/min/1.73 m^2^, the treatment can be continued even in the presence of lower eGFR values, namely CKD stage 4.

**Figure 1:**
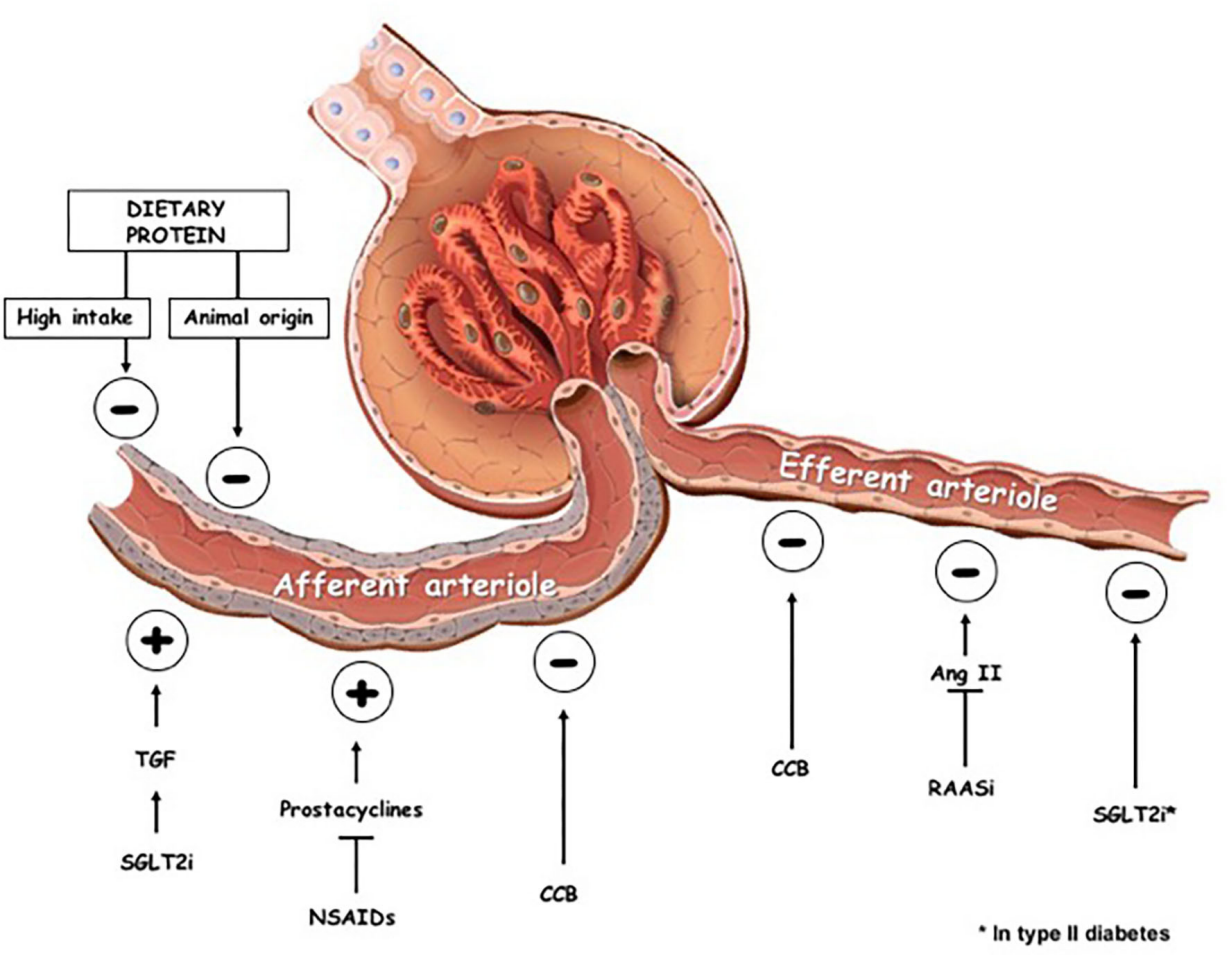
Effects on glomerular hemodynamics of dietary changes and pharmacologic interventions. TGF, tubuloglomerular feedback; SGLT2i, sodium-glucose-transporter 2 inhibitors; NSAIDs, non-steroidal anti-inflammatory drugs; CCB, calcium channel blockers; RAASi, renin-angiotensin-aldosteron system inhibitors; ⊕, vascular tone increase; ⊖, vascular tone decrease.

SGTL2i are not the only drugs able to modify glomerular hemodynamics: non-steroidal anti-inflammatory drugs increase the resistance of the afferent arteriole whereas RAAS inhibitors reduce resistance of efferent arteriole (11) (Figure 1). In addition, calcium channel blockers induce vasodilation of both the afferent and efferent arteriole, which is greater at the former than at the latter site (12) (Figure 1).

Besides drugs, significant changes of renal hemodynamics are also modulated by the amount and quality of protein intake (Figure 1).

Indeed, the role of diet in managing CKD has recently been sharply underlined by the new KDOQI guidelines on nutrition in kidney disease which extended the indication to prescribe a protein-restricted diet to delay ESKD in CKD patients since stage 3 (graded evidence level 1A) (13).

The combination between these two approaches is therefore appealing, but unfortunately, no data exist about the effects of SGLT2i in patients on low-protein diets vs. patients on unrestricted protein intake. Furthermore, we do not know if the effect of SGLT2i is modulated by salt intake, raising concerns about possible interferences or synergistic effects on renal outcomes.

The aim of this perspective paper is to review the similarities and differences of renal protection in the course of SGLT2i treatment or low-protein diet regimens and hypothesize their interferences, as a mean to call for studies on these emerging issue in the prevention and care of CKD.

## Effects of SGLT2i on Renal Hemodynamics and Kidney Function

### Tubuloglomerular Feed Back

Sodium glucose transporter 2 inhibitors act inhibiting the renal proximal tubule reabsorption of glucose and sodium (14). Murine models have shown that SGLT2 inhibitors are able to modulate renal hemodynamics, leading to reduction of glomerular hyperfiltration and urine albumin excretion. In fact, SGLT2 inhibitors reduce sodium reabsorption in the proximal tubule causing increased sodium delivery to the macula densa which, in turn, stimulates the tubuloglomerular feedback (TGF). As a consequence, vasoconstriction of the afferent glomerular arteriole occurs and causes a decrease in intraglomerular pressure and renal blood flow (15). This hypothesis was initially postulated by Cherney (16) in a cohort of young adults with Type 1 diabetes who underwent empagliflozin therapy for 8 weeks and was later confirmed in a murine model using multi-photon microscopy (17).

### Other Hemodynamic Effects

Despite the prevailing view that SGLT2i are able to restore pre-glomerular arteriolar resistance, Van Bommel et al. (18) observed that dapagliflozin acted lowering efferent arteriolar resistance in type 2 diabetes. In this context, a randomized, double-blind trial compared hemodynamic effects of gliclazide vs. dapagliflozin over 3 months in type 2 diabetic patients with eGFR > 60 ml/min/1.73 m^2^ and overt proteinuria. Dapagliflozin reduced GFR, filtration fraction and kidney vascular resistances through vasodilation of the efferent arteriole.

An increase in prostaglandin production has been reported in patients treated with dapagliflozin; prostaglandin release may cause post-glomerular arteriolar dilation, preventing TGF-mediated pre-glomerular vasoconstriction (19). This pattern resembles what happens reducing the protein intake (20) and, probably, sodium intake (21).

### Modulation of the Effect on Renal Hemodynamics by Type of Diabetes

There is no clear explanation regarding the discrepancy about the renal effects of SGLT2i in Type 1 or Type 2 diabetic patients but the possible causes are many. In hyperfiltrating, young type 1 diabetic patients, pre-glomerular resistance is low and the tubuloglomerular feedback, activated by empagliflozin, may cause constriction of the afferent artery via adenosine release (22), thus reducing intra glomerular pressure and glomerular filtration rate. Conversely, in older type 2 diabetic patients, glomerular hyperfiltration is less prominent and the potential for pre-glomerular vasoconstriction is limited due to arterial stiffness.

Even if renin-angiotensin-aldosterone system (RAAS) blockers should be included early in the treatment of all diabetic patients, especially at the appearance of proteinuria, it is possible that patients with type 2 diabetes are more often treated by these drugs at higher doses, for their anti-hypertensive effect (18).

### Effects on GFR and Albuminuria

From a clinical point of view, several studies describe an initial fall of eGFR (3, 4), followed by a raise and subsequent stabilization in the long term: this eGFR pattern has been called the “check-mark” sign (√) (23).

The drop in eGFR is probably one of the main mechanisms of nephroprotection and is rapidly reversible after discontinuation of therapy (15). Notably, this reduction of GFR following SGLT2i therapy is also observed in diabetic individuals without hyperfiltration and in patients with non-diabetic glomerular disease (24, 25), suggesting that it is not just due to an improved glycemic control.

In the long-term, treatment with SGLT2i is associated with a reduction of albuminuria and a slower decline of GFR when compared to placebo (26). These endpoints were achieved independently of changes of blood pressure or glucose control; indeed, the reduction of albuminuria with SGLT2i was associated to the reduction of intraglomerular pressure (15, 27).

Notably, the composite endpoint of retarding progression of kidney disease, expressed as doubling of serum creatinine or development of overt albuminuria, was also reached in a study conducted in with overt diabetic kidney disease (15).

### Autophagy

Recently, restoration of autophagy, a cell defense mechanism, was suggested as a contributor to the renoprotective effects of SGLT2i (28). Autophagy is a self-degradative process that occurs to save energy sources in conditions of nutrient deprivation. Autophagy allows disposing misfolded or aggregated proteins and damaged organelles, such as mitochondria, endoplasmic reticulum and peroxisomes, by pro-inflammatory or pro-oxidative stress.

An impairment of the kidney's autophagic capacity in conditions of increased oxidative stress has been shown to contribute to renal injury in type 2 diabetes (29, 30). The production of proinflammatory cytokines stimulated by cellular stress leads to cellular dysfunction and loss, and ultimately to inflammation and fibrosis (31, 32).

In the course of diabetes, autophagy dysregulation is attributed to an impaired nutrient deprivation signaling (SIRT1/AMPK) in podocytes and tubular cells (33–35). Moreover, diabetes may also impair the adaptive actions of hypoxia-inducible factors (36, 37).

SGLT2i appear to enhance autophagic capacity of kidney tissue and mitigate oxidative stress, through induction of hypoxia-like transcriptional patterns that enhance SIRT1/HIF-2a signaling (38). These molecular effects are peculiar of SGLT2i and are distinct from other glucose-regulating agents (28).

## Effects of Dietary Manipulation on Renal Hemodynamics and Kidney Function

### Hemodynamic Effects of Protein Restriction

Glomerular filtration is influenced by dietary protein intake in health and disease. As underlined by the recent KDGO guidelines, low-protein diets are a mean to reduce the progression of chronic nephropathies, in addition to the metabolic control of urea and phosphate levels and acidosis.

The effects of a low-protein diet have indeed many similarities with those of SGLT2i.

In animal models of CKD induced by subtotal nephrectomy, a low-protein diet is able to reduce hyperfiltration and hypertrophy in the remaining nephrons (39).

The reasons of this effect are manifolds: an increased glomerular filtration of amino acids and the consequent increased proximal tubular reabsorption increases sodium reabsorption in the proximal tubule and a decreased sodium delivery to the distal nephron. This, in turn, inhibits the tubuloglomerular feedback, lowering the resistance of the afferent arteriole, thus allowing glomerular hyperfiltration.

In CKD patients, a fall of GFR is observed in the first weeks of protein restriction; afterwards, however, kidney function stabilizes and the disease tends to progress slower than in patients on an unrestricted diet. This observation, derived from the classic MDRD trial, is consistent with an initial reduction of hyperfiltration followed by a slower CKD progression and is comparable to the “check-mark” sign (√) described in the course of treatment with SGLT2i, as mentioned above (23).

The quality of dietary proteins modulates glomerular hemodynamics and the perm-selectivity of the glomerular basement membrane. An acute load of animal proteins determines an increase not only in GFR and plasma renal flow, but also in the permeability of the glomerular basement membrane to albumin, in the absence of modification of the filtration fraction (39). This suggests a decrease in pre-glomerular vascular resistances. Conversely, an acute load of vegetable proteins (soy) is not associated with the aforementioned changes (40, 41). Moreover, subjects undergoing a plant-based diet, both in experimental as well as observational studies in vegan communities, usually display a lower GFR compared to subjects on a diet containing the same amount of mixed or animal-derived proteins (42).

### Effect of the Quality of Dietary Proteins

It has been hypothesized that the effect of plant-based diets is mediated by the lower serum concentrations of IGF-1 in vegans-vegetarians compared to omnivorous.

Recently, it has been reported that an omnivorous diet is associated with a higher GFR compared to a vegan diet, while a lacto-ovo-vegetarian diet was associated to an intermediate GFR value (43). In other words, the glomerular “reserve,” associated with the vasodilation capacity of the afferent arteriole, is not elicited by vegetable proteins, a mechanism probably, at least in part, mediated by phytoestrogens. Indeed, serum concentrations of isoflavones increase during a soy-based diet and inversely correlate with urinary albumin to creatinine ratio (44). In addition, soy proteins, as well as other plant derived proteins, may have anti-inflammatory properties, modulating interleukin 6, tumor necrosis factor alpha and nuclear factor-kB, displaying also an antioxidant activity at the renal level (45). In animal models, a soy-based diet may also reduce RAAS activity, decreasing resistance at the afferent arteriole and decreasing proteinuria (46). Finally, plant-based regimens have been shown to reduce cardiovascular risk (47–49). These data supported a plant-based diet for nephroprotection in healthy individuals as well as in renal patients (41), in particular with mild proteinuria and diabetic nephropathy (50, 51).

### Effect of Dietary Proteins on Tubulo-Glomerular Feedback and on the Hormonal Milieu

In diets rich in animal proteins, the inhibition of the tubuloglomerular feedback, the increase in glucagon secretion and the local production of vasodilatory prostacyclin have been called as explanations of hyperfiltration and increased glomerular permeability (52, 53). Indomethacin, an old, non-specific, antiproteinuric drug, reduces proteinuria in human and animal studies, supporting a major role of prostaglandins in the pathogenesis of hyperfiltration and proteinuria (54, 55).

In murine models, an isocaloric, protein restricted diet for 6 weeks decreases mitochondrial ROS production in the kidney (56). Low-protein diets may exert an anti-fibrotic effect through down-regulation of transforming growth factor-β (TGF-β) in mesangial cells (57).

In addition, animal models have shown that low-protein diets may reduce tubular cell damage, tubulointerstitial oxidative stress and apoptosis by decreasing the accumulation of abnormal mitochondria. This finding could be explained by the restoration of autophagy; furthermore, low protein diets reduce the mammalian target of rapamycin complex 1 (mTORC1) activity, promoting autophagic capacity (58, 59).

## Potential Synergy Between SGLT2i and Low-Protein Diets

So far, no study evaluated whether the type of diet, and its protein content, has a synergistic, neutral or counteracting effect on the action of SGLT2i. The main point is the site and mechanism of action.

In the case of RAAS inhibitors, the synergy between reduced intake of animal proteins has been investigated (60, 61). Conversely, if a competitive action of SGLT2i and protein restriction at the same level exists, the addition of a low-protein diet to SGLT2i therapy may not result in an additive effect.

The baseline protein intake may be important: a diet relatively rich in protein and restricted in lipids and carbohydrates is often recommended in diabetic patients. However, a high protein diet, especially if of animal origin, induces glomerular hyperfiltration that could blunt the favorable effects of SGLT2i. In this context, at least restoring a normal protein intake may allow setting a favorable stage for SGLT2i activity. Indeed, the recent Nutritional Guidelines in CKD (K-DOQI 2020) recommend normalizing the protein intake (0.8 g of proteins/Kg of ideal body weight/day) in diabetic patients (13). This strategy, overall, is expected to result in a reduction of the actual protein intake.

Furthermore, beyond hemodynamic effects, plant based, moderately protein-restricted diets have several additional advantages: they decrease the acid load (62) and phosphate intake, this easing the management of CKD-MDB (63). Moreover, the partial restoration of autophagy shared by low-protein diet and SGLT2i, may amplify the nephroprotective effects (Table 1).

**Table 1:** Beneficial effects of sodium-glucose-transporter 2 (SGLT2) inhibitors and low-protein/plant-based diets.

**Effect**	**SGLT2 inhibitors**	**Low-protein/plant-based diet**
Decreased sodium proximal tubular reabsorption	+ (14)	+ (39)
Restoration of the tubuloglomerular feedback	+ (15–17)	+ (39)
Reduction of glomerular hyperfiltration	+ (15)	+ (39)
Restoration of autophagy	+ (28, 38)	+ (58)
Slowing of CKD progression	+ (15, 26)	+ (41)
Reduction of unfavorable cardiovascular outcomes	+ (1, 2, 4)	+ (47–49)
Reduction of proteinuria	+ (26)	+ (44, 46)
Reduction of the acid load	-	+ (62)
Reduction of phosphate load	-	+ (63)

*Present: +. Absent: - (reference)*.

Conversely, in diabetic patients, insulin resistance or sub-optimal glycemic control could compromise the adaptation to protein restriction, increasing the risk of protein energy wasting (64). Of note SGLT2i induce a loss of up to 300 Kcal/day, which can enhance protein catabolism. Under these conditions, nutritional management should be careful and a plant-based diet, with normal protein intake (0.8-g of proteins/Kg/day), may be a nutritionally safe option, possibly enhancing nephroprotection.

## Conclusions

**Figure 2:**
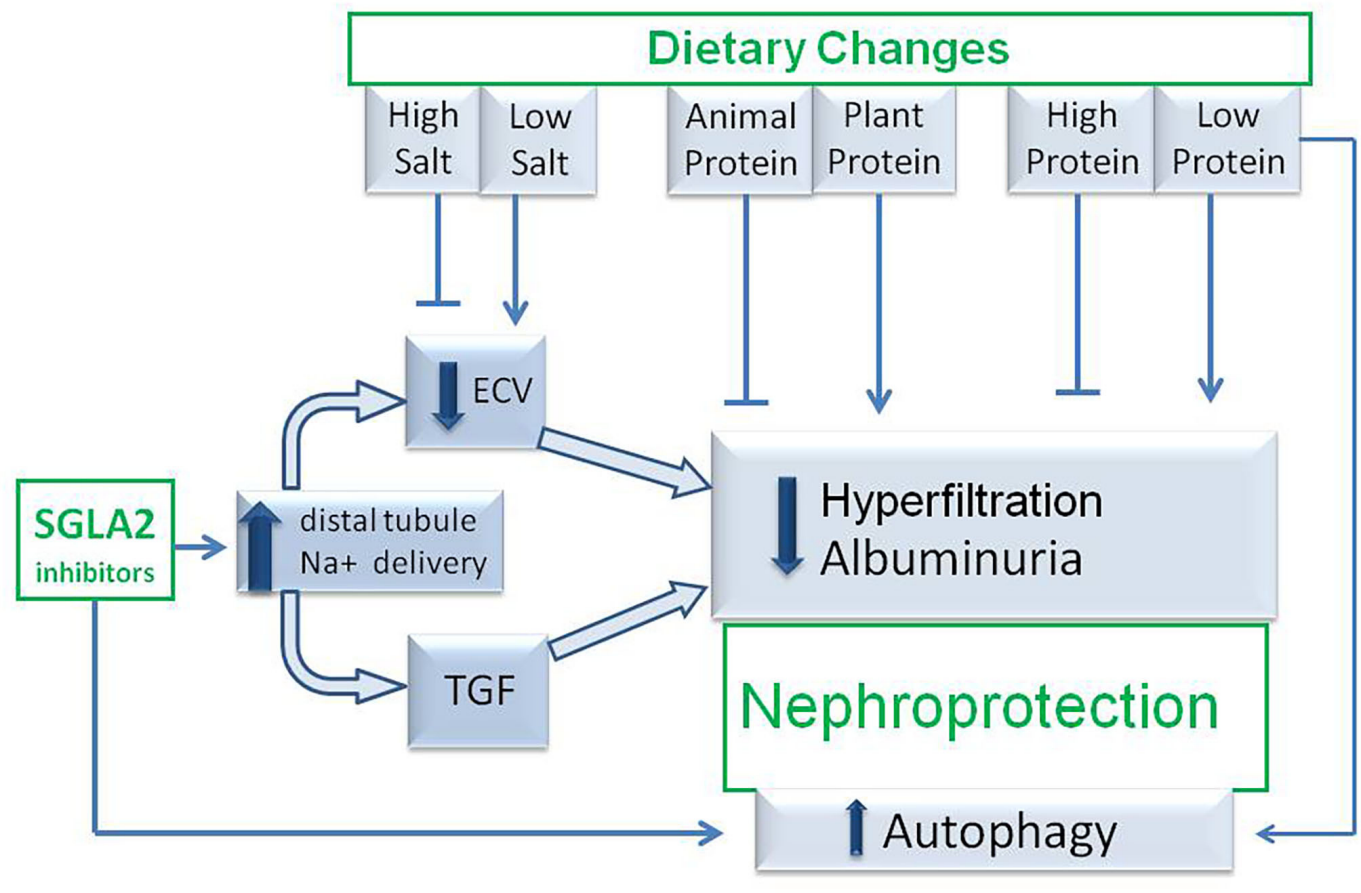
Hypothetic mechanisms of nephroprotection occurring during association between SGLT2 inhibitor therapy and medical nutritional therapy. TGF, tubuloglomerular feed-back; SGLT2i, sodium-glucose-transporter 2 inhibitors; ECV, extracellular volume.

SGLT2i and low-protein and/or vegetarian diets offer interesting opportunities of nephroprotection in both diabetic and non-diabetic CKD patients and they share some mechanisms of nephroprotection (Figure 2). Even if the pleiotropic effects of protein-restricted and plant-based diets may be expected to exert a favorable effect on top of a SGLT2i therapy, further studies should be targeted at assessing whether or not patients receiving a combined therapy have additional benefits in terms of nephroprotection and, if so, which dietary regiments have the safest and most efficient profile.

## Author Contributions

AC and GBP: conceptualization, original draft preparation, writing, review and editing. DG, DM, CD'A, and MT: writing, review and editing. All authors contributed to the article and approved the submitted version.

## Conflict of Interest

The authors declare that the research was conducted in the absence of any commercial or financial relationships that could be construed as a potential conflict of interest.
